# Tailoring magnetic properties of electrodeposited thin films of the molecule-based magnet Cr_5.5_(CN)_12_ 11.5H_2_O

**DOI:** 10.1186/1556-276X-7-232

**Published:** 2012-04-24

**Authors:** Helena Prima-Garcia, Eugenio Coronado, Juan P Prieto-Ruiz, Francisco M Romero

**Affiliations:** 1 Instituto de Ciencia Molecular, Universidad de Valencia, Catedrático José Beltrán 2, Paterna (Valencia), 46980, Spain

**Keywords:** Magnetic materials, Thin films, Molecule-based magnet, Prussian blue, MOKE

## Abstract

This paper reports on molecular-based magnetic thin films of Prussian blue analogues (PBA) with high critical temperatures composed of mixed-valence chromium cyanides. The thin films of PBA were synthesized by means of electrodeposition technique. Morphology and magnetic study are presented in a function of electrochemical deposition conditions. We present the electrochemical methods as a promising and effective tool for preparing molecular-based magnetic thin films of Prussian blue analogue.

## Background

The family of Prussian blue analogues (PBA) of general formula *C*_c_*A*_a_[*B*(CN)_6_]_b_·nH_2_O (*C*, alkali cation; *A* and *B*, transition metal ions) are molecule-based materials with interesting magnetic properties.

Electrochemical methods have been extensively used to fabricate high-quality films of molecule-based materials with interest in molecular spintronics such as PBAs [1-3].

Electrodeposition is considered as a real alternative to physical deposition techniques, such as evaporation, sputtering, and molecular beam epitaxy, in order to provide a simple and cost-effective technology for the preparation of high-quality films and multilayers [4,5]. Electrochemical methods are intrinsically fast and compatible with patterning and large-scale production [6] with the possibility of working in wet non-vacuum conditions.

In this way, electrodeposition is presented as one of the simplest and cheapest processes available for the fabrication of thin films [7] being of great interest in possible industrial applications.

In this work, we report on the fabrication and characterization of Prussian blue analogue thin films obtained by electrodeposition, showing this technique as a promising and effective tool for preparing high-quality thin films of this molecule-based magnet with great perspectives in the field of molecular spintronics.

## Methods

### Preparation of the films

All reagents were purchased from Sigma-Aldrich (St. Louis, MO, USA) and used without further purification. The electrochemical synthesis of the PBA Cr_3.5_^II^[Cr^III^(CN)_6_[Cr^II^(CN)_6_·11.5H_2_O has been described in previous works [8]. An aqueous solution containing K_3_[Cr(CN)_6_ (5 mmol) and CrCl_3_ (7.5 mmol) was reduced at a fixed potential (*E* = −0.88 V vs Ag/AgCl reference electrode) on a substrate of Mylar or glass (dimension 5 × 10 mm) coated with an evaporated Au layer of 100-nm thickness. This substrate was used as the working electrode. A Metrohm Autolab potentiostat (Kanaalweg, Utrecht, The Netherlands) in coulometry mode was employed for depositing the transparent films with a Pt wire as a counter electrode. After preparation, films were rinsed with deionized water and dried at room temperature. Films of different thicknesses were obtained by varying the time of deposition. The thicknesses were determined using an Ambios Technology XP-1 profilometer (Milpitas, CA, USA) placed on a vibration isolation table.

### AFM -study

A Nanoscope Multimode (Veeco Instruments Inc., Plainview, NY, USA) atomic force microscopy in tapping mode operation was used in the morphological study of the electrodeposited films. Root mean square (RMS) roughness and average particle size were determined using WSx4.0 Develop 13.0 software [9], developed by Nanotec Electronics S.L. (Tres Cantos, Madrid, Spain).

### MOKE measurements

The magneto-optical characterization was performed with a self-made Kerr magnetometer. A He-Ne laser with a wavelength of 633 nm and an output power of 12 mW was used as the light source producing a nearly linearly-polarized light beam. This beam was passed through a Glan-Laser calcite polarizer (Karl Lambrecht Corporation, Chicago, IL, USA) with an extinction coefficient of 10^−5^, which allows working with both s-polarized (electric field perpendicular to the plane of incidence) and p-polarized (electric field parallel to the plane of incidence) configurations. The design of the electromagnets and the cryostat allows the use of longitudinal, polar, and transverse geometries. The external magnetic field range in the polar configuration is ±150 mT.

## Results and discussion

By reducing [Cr(H_2_O)_6_^3+^ at a fixed potential of *E* = −0.88 V in an aqueous solution containing [Cr(CN)_6_^3−^ anions, it was possible to obtain thin films of Cr_x_(CN)_6_·zH_2_O [10] (1). Thus, an electroactive transparent film is formed *in situ* by a reaction of the [Cr(H_2_O)_6_^2+^ species with the hexacyanometalate anion on the surface electrode resulting in the formation of the insoluble PBA. By changing the electrodeposition time in coulometry, it was possible to tune the thickness of the electrodeposited films. The relationship between the time of deposition and the film thickness was found to be nearly linear (Table 1). The use of this electrochemical method allowed us to fabricate films with thicknesses ranging from 1,500 nm for 100 s deposition time to 40 nm for films with a deposition time of 1 s.

**Table 1:** Morphological parameters of different thin films of 1

**Time of deposition (s)**	**Thickness (nm)**	**Average particle size (nm)**	**RMS roughness (nm)**	**Coercive field**^**a**^**(mT)**
100	1,500 ± 100	600 ± 100	58	32 ± 5
50	450 ± 30	320 ± 80	37	49 ± 5
25	250 ± 10	180 ± 30	18	76 ± 5
10	80 ± 10	170 ± 40	10	85 ± 5
2	50 ± 10	120 ± 20	10	33 ± 5
1	40 ± 10	90 ± 20	8	10 ± 5

^a^ At 190 K.

Optical absorption spectra of the electrodeposited films of 1 [11] and attenuated total reflectance infrared spectra (ATR-IR) have been studied somewhere else. The ATR-IR spectra showed for all the different thicknesses a principal band located at 2,186 cm^−1^ corresponding to the cyanide stretching vibration of the [Cr^III^(CN)_6_^3−^ anion in concordance with the previous results [10]. The IR frequency associated with this specie is independent of the film thickness [11], indicating that the composition of the film is unaltered as the thickness is decreased.

**Figure 1:**
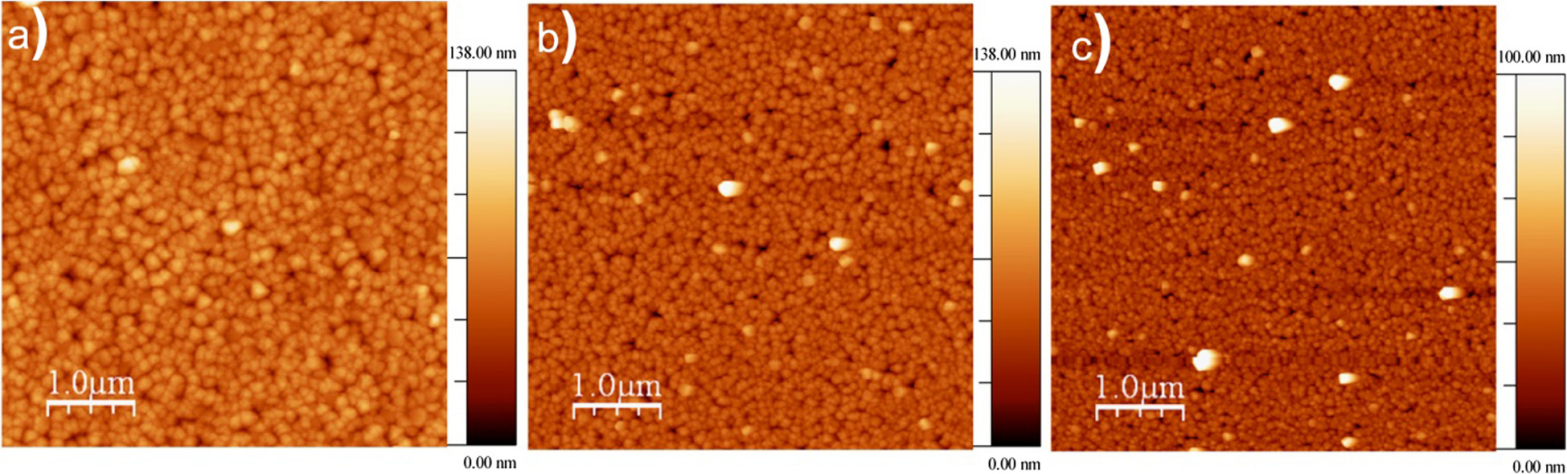
**AFM topography images.** Surface of thin films of **1** obtained after different times of electrodeposition: (**a**) 10 s (80-nm thickness), (**b**) 2 s (50-nm thickness), and (**c**) 1 s (40-nm thickness).

In order to characterize the morphology of the films of 1, an atomic force microscopy (AFM) study was performed (Figure 1). In this case, only the images corresponding to the thinner films obtained so far are showed. The images were acquired in tapping mode, with a 5 × 5-μm scan sizes for films with different deposition times. For the thicker films, it is possible to distinguish a polycrystalline structure composed of pyramidal particles of different sizes [11]. This type of morphology has been observed in the epitaxial electrodeposition of Prussian blue on Au(110) surface [12]. For the thinner films, the average particle size (Table 1) increases from a value of 90 nm (lateral size) for the film prepared after 1-s electrodeposition (40-nm thickness) to a value of 170 nm for the film of 80 nm. During the initial (nucleation) stages of the electrodeposition process, the reaction takes place at the naked surface of the electrode, and its kinetics is only limited by diffusion of the species, resulting in very small particles that cover homogenously the electrode surface. At longer deposition times, the intensity of the electrochemical current decreases, indicating a slower kinetics that results in the growth of the particles as pyramidal microcrystals as has been studied at thicker films of sample (1) [11]. The roughness of the film surface shows a slight increase with the deposition time, passing from a value of 8 nm for the 40-nm thickness film to a value of 10 nm for the 80-nm thickness film.

The magnetic properties of the films at different thickness have been studied somewhere else [11] exhibiting negligible changes on the magnetic properties when changing the film thickness.

Magneto-optical Kerr effect (MOKE) measurements were performed in order to probe directly the magnetic properties of the different thin films described in Table 1. A source of linearly polarized light of high wavelength (*λ* = 633 nm) and low power (12 mW) was used in order to prevent any photomagnetic effect. The Kerr rotation (*θ*_k_) of the films, directly proportional in a first approximation to the magnetization of the samples, was recorded as a function of the applied magnetic field for different temperatures. The first striking result from the MOKE study is that a relatively large hysteresis loops appeared just below *T*_c_ for all the films measured in polar configuration (magnetic field perpendicular to the surface sample). The difference between MOKE and other magnetometer like superconducting quantum interference device (SQUID) is probably due to the local surface character of the MOKE technique, which is more sensitive to subtle variations in the morphology of the sample. Also, it has to be considered that the MOKE signal at a given energy of the incident light depends on the joint magneto-optical density of states.

**Figure 2:**
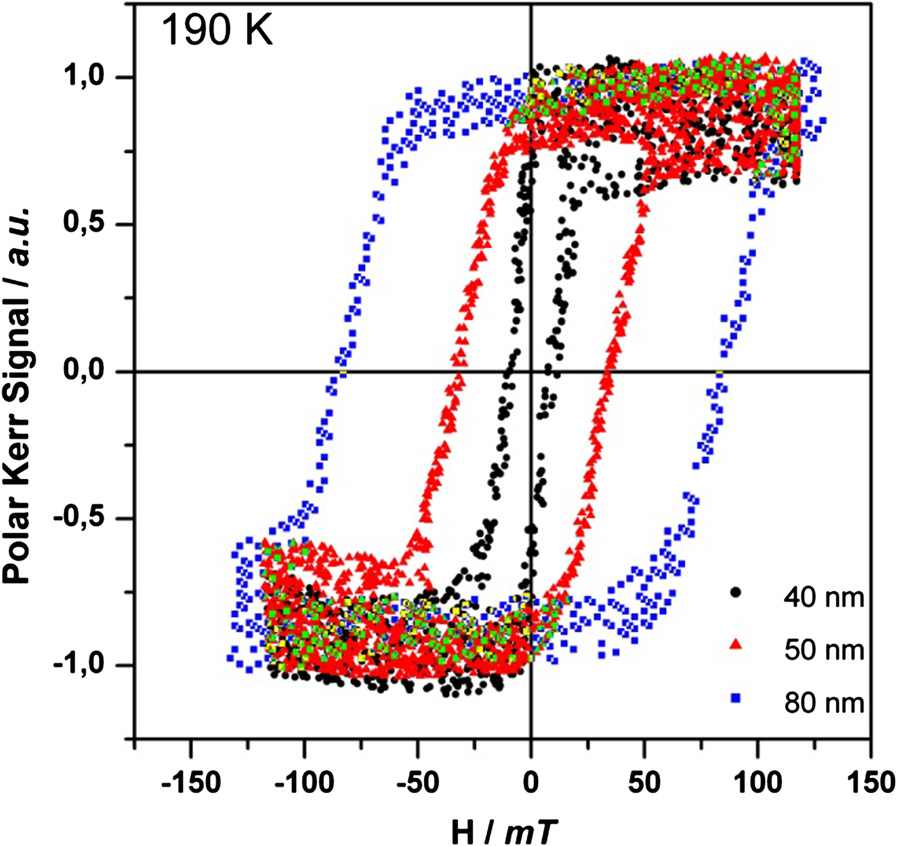
**Normalized MOKE hysteresis loops.** Obtained in polar configuration for three thin films of **1** of various thicknesses.

**Figure 3:**
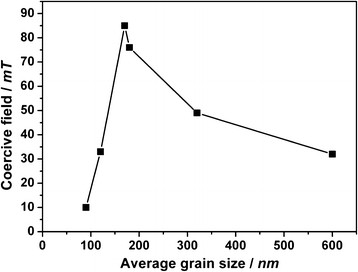
**Coercive field.** As a function of the average grain size of thin films of **1** shown in Table 1.

The magneto-optical response at 190 K of the thinner films of **1** with thicknesses ranging from 80 to 40 nm is depicted in Figure 2. In contrast with the SQUID measurements, where it was found to be independent of the film thickness, here, the coercive field suffers an important change in value when decreasing the film thickness (Table 1). This tendency has been studied [11]. In Figure 3, the coercive field is showed as a function of the mean grain size. When the particle size is reduced, the coercive field increases, passing through a maximum and then decreasing again. The magnetization reversal process for the particles composing the film is different in each region of the curve in Figure 3. The thicker films with a bigger grain size consist of relatively large multi-domain magnetic particles, whereas in the thinner films, the particles approach the single-domain limit, resulting in an increase in coercivity. Such a particle-size effect has been previously described in other molecule-based materials [13-15].

In consequence, there is an increase in coercive field with the reduction of the grain size until this value reaches a maximum, in our case 85 mT for a grain size of 170 nm; underneath this value, the physical processes associated to the magnetization reversal in smaller grains produce a reduction in coercive field.

## Conclusions

In summary, it has been demonstrated in this work that it is possible to tune the thickness of electrodeposited films of Prussian blue analogues by changing the electrodeposition time. Therefore, it is possible to reach the nanometer scale in thin films of PBA by reducing the electrodeposition time to few seconds. Thus, the morphology of the films of PBA is also modified varying the thickness of the film by a change in the average grain size. These thinner films possess a smoother surface consisting in homogeneous particles of smaller size. Interestingly, as the thickness of the film is reduced, its magnetic properties are considerably improved (higher coercivity and squareness of the hysteresis loops) until a limit is reached beneath this limit; the coercivity is reduced surely until the superparamagnetic limit.

Consequently, the utility of electrochemical methods has been also demonstrated in order to obtain in a simple, fast, and reproducible way high-quality thin films of PBA which are molecule-based magnets with very interesting magnetic properties and possible applications in molecular spintronic.

In view of the versatility of these materials, which include the easy tuning of the magnetic (nature of the magnetic ordering, anisotropy, coercivity, and critical temperature) and electronic (redox potential, energy gap) properties by simply varying the nature of the metal centers, future work will focus on the fabrication and MOKE characterization of PBA-based magnetic multilayers to study proximity effects as well as on the use of these films as spin injector of all-molecular spin valves.

## Competing interests

The authors declare that they have no competing interests.

## Authors' contributions

JPPR carried out the preparation of the samples and their characterization with AFM. HPG carried out the characterization of the samples by MOKE and SQUID, has also written the paper, and supervised the work of JPPR. EC and FMR participated in the supervision of all the study. All authors read and approved the final manuscript.
